# Spatial Mutual Information Based Hyperspectral Band Selection for Classification

**DOI:** 10.1155/2015/630918

**Published:** 2015-03-30

**Authors:** Anthony Amankwah

**Affiliations:** Computer Science Department, University of Ghana, Legon, Ghana

## Abstract

The amount of information involved in hyperspectral imaging is large. Hyperspectral band selection is a popular method for reducing dimensionality. Several information based measures such as mutual information have been proposed to reduce information redundancy among spectral bands. Unfortunately, mutual information does not take into account the spatial dependency between adjacent pixels in images thus reducing its robustness as a similarity measure. In this paper, we propose a new band selection method based on spatial mutual information. As validation criteria, a supervised classification method using support vector machine (SVM) is used. Experimental results of the classification of hyperspectral datasets show that the proposed method can achieve more accurate results.

## 1. Introduction

Hyperspectral imaging consists of a large number of closely spaced bands that range from 0.4 *μ*m to 2.5 *μ*m [1]. The high dimensionality in hyperspectral imagery makes it useful for many applications such as agriculture, medicine, and surveillance. However, the high dimensionality of hyperspectral data leads to high computational cost and can contain redundant information. Thus, there is need to select the relevant bands to reduce computational cost and data storage while maintaining accuracy.

Band selection or feature extraction can be used to reduce hyperspectral data. In band selection, a representative subset of the original hyperspectral information is selected [2, 3]. Feature extraction involves the reduction of the original information by transforming the initial information [4, 5]. In hyperspectral imaging band selection is preferred since original information is preserved, whereas in feature extraction the original and required information may be distorted [6]. In pixel classification a good band selection method can not only reduce computational cost but also improve the classification accuracy.

Typically, in band selection, the similarity space is defined among hyperspectral bands after converting the image bands into vectors, where a dissimilarity measure is defined based on the information measures such as mutual information between a pair of vectors. The vectors are then clustered into several groups based on their dissimilarity. In our work, we use hierarchical clustering [7] in the dissimilarity space. In the end, for each of the clusters, a band is selected to represent each cluster. The dissimilarity metric used will influence the shape of the clusters, as some elements may be close to one another according to one distance and farther away according to another.

The maximization of mutual information criterion postulates that mutual information is maximal, when image bands are similar. Mutual information has been demonstrated to be a very general and powerful similarity metric, which can be applied automatically and very reliably, without prior preprocessing, on a large variety of applications [8]. Mutual information treats all pixels the same during signal matching regardless of the position and usefulness of the pixel in the image. However, it does not incorporate useful spatial information which is a drawback.

In this work, we propose spatial mutual information which combines mutual information and a weighting function based on absolute difference of corresponding pixels as the dissimilarity metric and hierarchical clustering to select the bands considered most relevant. We tested our proposed algorithm on two hyperspectral AVIRIS datasets with 220 and 204 band images, respectively, and their corresponding ground truths. The experimental results show that using our proposed dissimilarity metric provides a more suitable subset of bands for pixel classification.

## 2. Dissimilarity Measures

The independence of bands is one of the main factors used to select a subset of image bands for pixel classification. Dissimilarity measures are used to quantify the degree of independence of image bands. Information measures such as mutual information are widely used to measure the correlation between information from different sensors.

### 2.1. Mutual Information

If *X* and *Y* are two image bands, the mutual information MI can be defined by

(1) $$\begin{array}{c} \text{MI}=H\left(X\right)+H\left(Y\right)-H\left(X,Y\right), \end{array}$$

where *H*(*X*) and *H*(*Y*) are the Shannon entropies [8] of *X* and *Y*, respectively, and *H*(*X*, *Y*) is the Shannon entropy of the joint distribution of *X* and *Y*. *H*(*X*) is defined as

(2) $$\begin{array}{c} H\left(X\right)=\overset{N}{\underset{i=1}{\sum }}p_{X}\left(i\right)\log p_{X}\left(i\right), \end{array}$$

where *p*_*X*_(*i*) is the probability distribution.

Equation (1) contains the term −*H*(*X*, *Y*), and it means minimizing joint entropy is increasing mutual information. Since generally joint entropy increases with increasing dissimilarity, the mutual information decreases with increasing dissimilarity. In other words, if image bands are similar the amount of mutual information they contain about each other is high.

In our work, the histogram method was used to estimate the MI between image bands; thus,

(3) $$\begin{array}{c} \text{MI}=\frac{1}{E}\sum_{x}\sum_{y}\text{His}{\text{t}}_{xy}\left(X,Y\right)*\log\left(\frac{E*\text{His}{\text{t}}_{xy}\left(X,Y\right)}{\text{His}{\text{t}}_{x}\left(X\right)*\text{His}{\text{t}}_{y}\left(Y\right)}\right), \end{array}$$

where *E* is the number of entries. Hist_*x*_(*X*) and Hist_*y*_(*Y*) are defined as their histograms and Hist_*xy*_(*X*, *Y*) as joint histogram.

**Figure 1:**
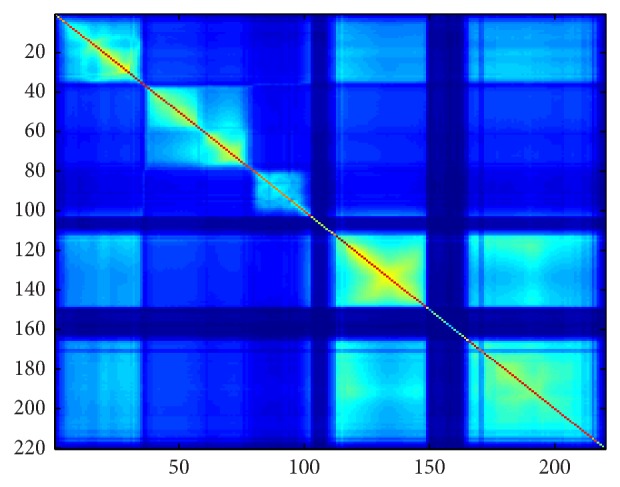
Dissimilarity matrix of hyperspectral image with 220 bands using MI.

Figure 1 shows the dissimilarity matrix of 220-band AVIRIS Indian Pines image scene using MI.

### 2.2. Spatial Mutual Information

**Figure 2:**
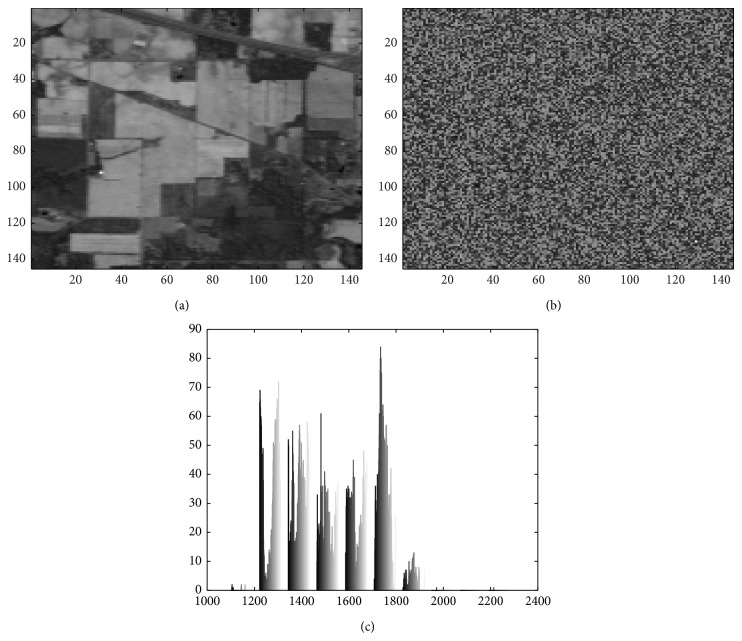
(a) 144th band of AVIRIS Indian Pines scene. (b) Randomized image in (a). (c) Histogram of images in (a) and (b).

We have extended MI to include spatial information. MI is estimated on a pixel to pixel basis, meaning that it takes into account only the relationships between corresponding individual pixels and not those of each pixel in the respective neighbourhood. As a result, much of spatial information inherent in images is not utilized. If an image band is reshuffled it will yield the same MI. Thus, the MI between Figure 2(a) and Figure 2(a) (itself) and the MI between Figure 2(a) and Figure 2(b) are the same.  Figure 2(c) is the histogram of image in Figure 2(a) or Figure 2(b).

Our proposed spatial mutual information (SMI) combines mutual information with a weighting function based on the absolute difference of corresponding pixel values. The absolute differences provide the spatial information. The sum of absolute difference can be considered as another similarity metric.

If *X* and *Y* are image bands the spatial mutual information is defined by

**Figure 3:**
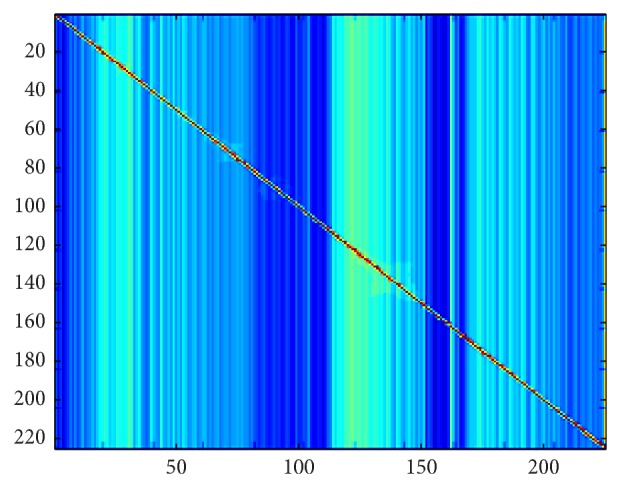
Dissimilarity matrix of hyperspectral image with 220 bands using SMI.

(4) SMI=1M∑x∑yDiff(X,Y)∗Histxy(X,Y)∗log⁡M∗Histxy(X,Y)Histx(X)∗Histy(Y),

where Diff(*X*, *Y*) is the weighting function based on the absolute difference of corresponding pixels.  Figure 3 shows the dissimilarity matrix of 220-band AVIRIS Indian Pines image scene using SMI.

## 3. Our Proposed Band Selection Algorithm

The goal of our algorithm is to select a subset of image bands that are independent as possible. The independence of selected bands increases the accuracy of classification of pixels [9]. We use the dissimilarity measure spatial mutual information to define a dissimilarity space as shown in Figure 3. Then, clustering is used to group bands according to the information they share. Finally, a band representing each cluster is selected for classification purposes.

Hierarchical clustering is used in this work. It is normally represented in tree structures with a nested set of partitions. The dissimilarity space is used to obtain a sequence of disjoint partitions. The distance between each pair of groups is used to decide how to link nested clusters in the consecutive levels of the hierarchy. One interesting characteristic of hierarchical methods is the fact that different linkage strategies create different tree structures. We use an agglomerative strategy in this work. That is, it starts with *m* initial clusters and, at each step, merges the two most similar groups to form a new cluster. Thus, the number of groups is reduced one by one [10].

In the end, bands are grouped according to the amount of information they share. In a final stage, a band representing each cluster is chosen, in such a way that the band selected will share as much information with respect to the other bands in the cluster.

## 4. Experiments and Results

In our experiments, datasets are used to evaluate the performance of the proposed method. The first dataset is the Airborne Visible/Infrared Imaging Spectrometer (AVIRIS) image taken over northwestern Indiana's Indian Pine test site, which has been widely used for experiments [11, 12]. The Indian Pine dataset is with the resolution of 145 × 145 pixels and has 220 spectral bands. There are 16 classes in total, ranging in size from 20 to 2455 pixels. The dataset is accompanied with a reference map, indicating the ground truth. The background class was not considered for classification. The Salinas dataset consists of 204 spectral bands with size of 217 × 512 pixels [13]. There are 16 classes in total ranging from 916 to 11721 pixels. The background area was not used for classification.

In this work, use the support vector machine (SVM) for classification. The SVM classifies data into two groups by constructing a hyperplane [14]. Intuitively, a good separation is achieved by the hyperplane that has the largest distance to the nearest training data point of two classes. Generally the larger the margin the lower the generalization error of the classifier. In this work, we use the multiclass SVM scheme, named one-versus-all. The one-versus-all scheme involves the division of an *N* number of classes dataset into *N* two-class cases. The radial basis function (RBF) is used as the kernel function in this experiment.

The pixels from every 16 classes are randomly separated into 55% and 45% as the training and testing data, respectively. For our experiment, 5,702 and 61107 pixels form the training data of the Indian Pines and Salinas datasets, respectively. The rest of the pixels for each dataset form the testing data. The ground truths of the Indian Pines and Salinas datasets are shown in Figures 5 and 6, respectively. The following lists show the classes of the Indian Pines and Salinas datasets, respectively.

*Indian Pines AVIRIS Ground Truth Classes*

1. Background
2. Alfalfa
3. Corn no Till
4. Corn-min Till
5. Corn
6. Grass-pasture
7. Grass-trees
8. Grass/Pasture-mowed
9. Hay-windrowed
10. Oats
11. Soybean no Till
12. Soybean min Till
13. Soybean-clean
14. Wheat
15. Woods
16. Building-Grass Tree-Drives
17. Stone-Steel Towers.

*Salinas AVIRIS Ground Truth Classes*

1. Background
2. Brocoli green weeds 1
3. Brocoli green weeds 2
4. Fallow
5. Fallow rough plow
6. Fallow smooth
7. Stubble
8. Celery
9. Grapes untrained
10. Soil vineyard develop
11. Corn senesced green weeds
12. Lettuce romaine 4 wk
13. Lettuce romaine 5 wk
14. Lettuce romaine 6 wk
15. Lettuce romaine 7 wk
16. Vinyard untrained
17. Vinyard vertical trellis.

**Figure 4:**
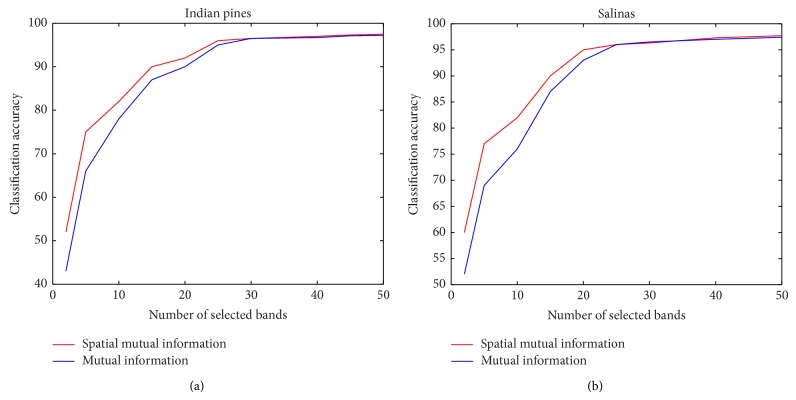
Classification results between our proposed algorithm and using mutual information to define the dissimilarity space for (a) Indian Pines dataset and (b) Salinas dataset.

**Figure 5:**
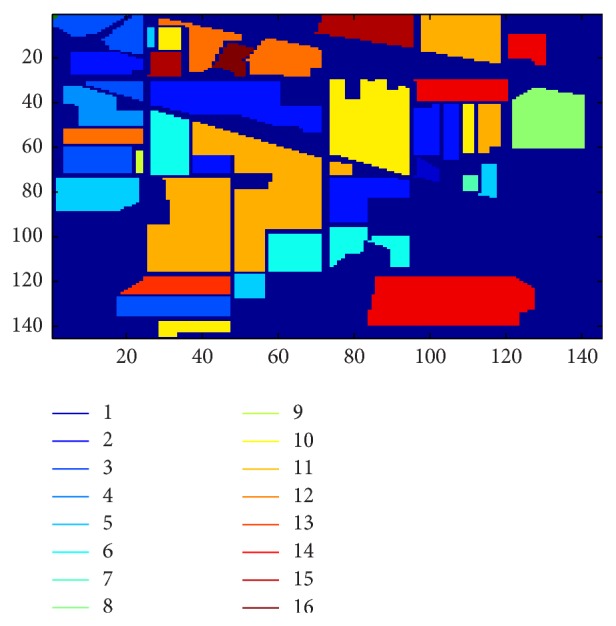
Ground truth for Indian Pines dataset.

**Figure 6:**
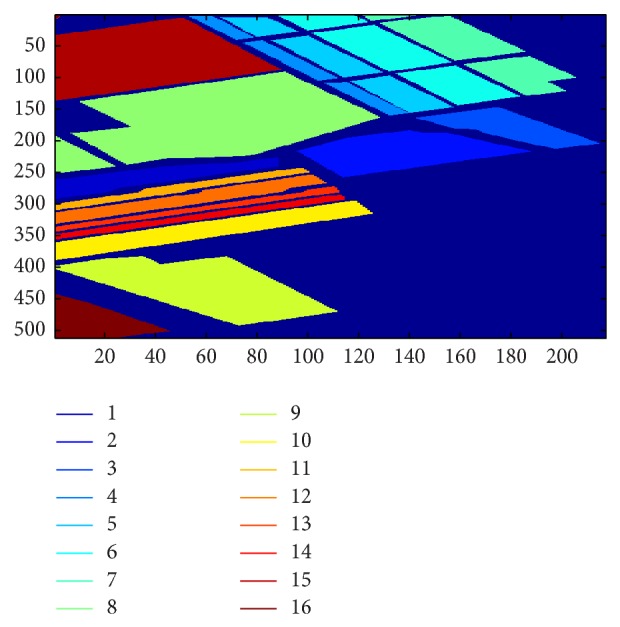
Ground truth for Salinas dataset.

We evaluated the overall accuracy which is the total number of correctly classified samples versus the number of samples. Figures 4(a) and 4(b) compare the classification accuracy using our proposed algorithm and one of the popular methods used for band selection [15–17], which has a similar configuration as in our proposed algorithm but MI is used to define the dissimilarity space as shown in Figure 1.

**Figure 7:**
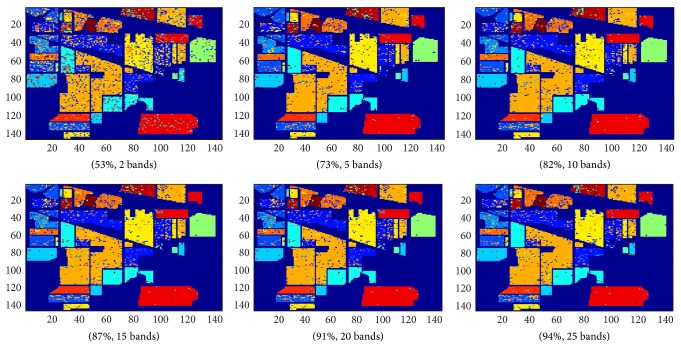
Classification results of Indian Pines dataset using our proposed algorithm.

**Figure 8:**
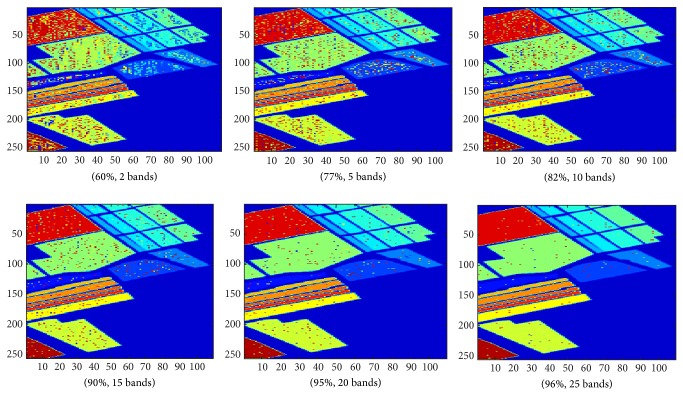
Classification results of Salinas dataset using our proposed algorithm.

The classification accuracy of our proposed algorithm is generally higher than using MI. For smaller numbers of band selection our proposed method is particularly more robust. The average classification accuracy for the Indian Pines dataset using number of bands selected from 2 to 10 for our proposed method and using MI is 70% and 65%, respectively. The average classification accuracy for the Salinas dataset using the same number of bands range for our proposed method and using MI is 73% and 67%, respectively. Figures 7 and 8 visualize the classification results of our experiment. The figures show that there is general improvement in classification accuracy with the increasing with number of bands selected.

## 5. Conclusions

In this paper, we propose a new hyperspectral band selection algorithm for pixel classification. The algorithm uses spatial mutual information to calculate the dissimilarity space for band selection. We compare our method to a state-of-the-art method where mutual information is used as the dissimilarity metric. The experiments demonstrate that our proposed method can achieve more accurate pixel classification results than using mutual information. In future, we will apply our proposed method to other large datasets and investigate optimization algorithms to reduce computational cost.
